# Outer membrane vesicles of *Porphyromonas gingivalis* trigger NLRP3 inflammasome and induce neuroinflammation, tau phosphorylation, and memory dysfunction in mice

**DOI:** 10.3389/fcimb.2022.925435

**Published:** 2022-08-09

**Authors:** Ting Gong, Qi Chen, Hongchen Mao, Yao Zhang, Huan Ren, Mengmeng Xu, Hong Chen, Deqin Yang

**Affiliations:** ^1^ Department of Endodontics, Stomatological Hospital of Chongqing Medical University, Chongqing, China; ^2^ Chongqing Key Laboratory of Oral Diseases and Biomedical Sciences, Chongqing Medical University, Chongqing, China; ^3^ Chongqing Municipal Key Laboratory of Oral Biomedical Engineering of Higher Education, Chongqing Medical University, Chongqing, China

**Keywords:** *porphyromonas gingivalis*, outer membrane vesicles, inflammasome, neuroinflammation, Alzheimer’s disease, tau

## Abstract

**Background:**

*Porphyromonas gingivalis* (Pg), the keystone pathogen in chronic periodontitis, is reported to initiate Alzheimer’s disease pathologies in preclinical studies. However, the specific mechanisms and signaling pathways acting on the brain still need to be further explored. Outer membrane vesicles are derived from Gram-negative bacteria and contain many virulence factors of bacteria. We hypothesized that outer membrane vesicles are an important weapon of *Porphyromonas gingivalis* to initiate Alzheimer’s disease pathologies.

**Methods:**

The outer membrane vesicles of *Porphyromonas gingivalis* (Pg OMVs, 4 mg/kg) or saline were delivered to 14-month-old mice by oral gavage every other day for eight weeks. Behavioral alterations were assessed by the open field test, Morris water maze, and Y-maze test. Blood–brain barrier permeability, neuroinflammation, tau phosphorylation, and NLRP3 inflammasome-related protein were analyzed.

**Results:**

Pg OMVs impaired memory and learning ability of mice and decreased tight junction–related gene expression ZO-1, occludin, claudin-5, and occludin protein expression in the hippocampus. Pg OMVs could be detected in the hippocampus and cortex three days after oral gavage. Furthermore, Pg OMVs activated both astrocytes and microglia and elevated IL-1β, tau phosphorylation on the Thr231 site, and NLRP3 inflammasome–related protein expression in the hippocampus. In *in vitro* studies, Pg OMV (5 µg/ml) stimulation increased the mRNA and immunofluorescence of NLRP3 in BV2 microglia, which were significantly inhibited by the NLRP3 inhibitor MCC950. In contrast, the tau phosphorylation in N2a neurons was enhanced after treatment with conditioned media from Pg OMV-stimulated microglia, which was attenuated after pretreatment with MCC950.

**Conclusions:**

These results indicate that Pg OMVs prompt memory dysfunction, neuroinflammation, and tau phosphorylation and trigger NLRP3 inflammasome in the brain of middle-aged mice. We propose that Pg OMVs play an important role in activating neuroinflammation in the AD-like pathology triggered by *Porphyromonas gingivalis*, and NLRP3 inflammasome activation is a possible mechanism.

## 1 Introduction

Alzheimer’s disease (AD) is the main cause of dementia and represents an enormous burden for the health economies. β-amyloid (Aβ) and tau phosphorylation, the main components of senile plaque and neurofibrillary tangles (NFTs), respectively, in the brain, are characteristic pathological hallmarks of AD (Braak and Braak, 1991).

Epidemiological studies propose that periodontal disease is a risk factor for AD (Sparks et al., 2012; Noble et al., 2014; Ryder, 2020). *Porphyromonas gingivalis* (Pg) is a Gram-negative bacterium known as a major pathogen of periodontal disease (Darveau et al., 2012). Many experimental studies conclude that Pg or its virulence factors could induce memory impairment and AD-related pathologies (Wu et al., 2017; Ding et al., 2018; Ilievski et al., 2018; Zhang et al., 2018; Dominy et al., 2019; Hayashi et al., 2019; Nie et al., 2019; Gu et al., 2020; Hu et al., 2020; Chi et al., 2021; Hao et al., 2022 Jiang et al., 2021; Qian et al., 2021; Su et al., 2021; Tang et al., 2021). Gingipains are toxic proteases secreted by Pg, it is reported that gingipains are neurotoxic *in vivo* and *in vitro*, causing detrimental effects on tau, a protein needed for normal neuronal function (Dominy et al., 2019), and gingipains could degrade tight junction proteins of human cerebral microvascular endothelial cells *in vitro* (Nonaka et al., 2022), suggesting that gingipains may be responsible for blood–brain barrier (BBB) damage. Lipopolysaccharide (LPS) is another virulence factor of Pg; Pg LPS can induce neuronal inflammation through the TLR4/NF-κB pathway (Zhang et al., 2018) and intracellular Aβ accumulation in neurons in a cathepsin B–dependent manner (Wu et al., 2017). However, the specific virulence factors, mechanisms, and signaling pathways acting on the brain still need to be further explored.

Outer membrane vesicles (OMVs) are gaining researchers’ attention. OMVs are secreted by Gram-negative bacteria; range from 20 to 250 nm in diameter; and carry bacterial LPS, protease, membrane receptor, DNA, RNA, and so forth (Toyofuku et al., 2019). It is suggested that OMVs could act as long-distance weapons and cause systemic disease, including AD (Amano et al., 2010; Schertzer and Whiteley, 2013; Singhrao and Olsen, 2018; Seyama et al., 2020; Wei et al., 2020; Nara et al., 2021). In fact, the majority of Pg gingipains are packaged and associated with Pg OMVs (Nara et al., 2021), and Pg OMVs could increase BBB permeability and degrade tight junction proteins in a human *in vitro* model (Pritchard et al., 2022). Thus, we aimed to explore the effect of Pg OMVs on AD pathologies.

Neuroinflammation is suggested to play a critical role in AD onset and progression (Heneka et al., 2015). IL-1β are elevated in brains of AD patients and can be associated with the onset and progression of AD (Alvarez et al., 1996; Griffin et al., 2000; Oprica et al., 2007; Deniz-Naranjo et al., 2008). The nucleotide-binding oligomerization domain-like receptor family, pyrin domain containing 3 (NLRP3), is an intracellular signaling molecule that senses many pathogen-, environmental-, and host-derived factors (Wen et al., 2013). Upon activation, NLRP3 binds to an apoptosis-associated speck-like protein containing a CARD (ASC), and ASC, in turn, interacts with procaspase-1, forming a complex termed the inflammasome. This results in the formation of the active caspase-1 p10/p20 tetramer, which then processes cytokine proforms, such as IL-1β and IL-18, to generate active molecules and mediates a type of inflammatory cell death known as pyroptosis (Schroder and Tschopp, 2010). The NLRP3 inflammasome is found to play an important role in microglial activation (Tan et al., 2013), and inhibition or knock-out of the NLRP3 inflammasome could reduce Aβ and tau phosphorylation *in vitro* and *in vivo* (Heneka et al., 2013; Ising et al., 2019; Beyer et al., 2020).

Given that the NLRP3 inflammasome is important in AD progression, it has become the focus of research, which may help to uncover the mechanism of AD. Recently Pg OMVs were reported to activate the NLRP3 inflammasome and induce IL-1β production in macrophages and monocytes (Cecil et al., 2017; Fleetwood et al., 2017).

The aim of this study was to explore whether Pg OMVs can trigger the NLRP3 inflammasome and neuroinflammation in the hippocampus, thus potentially inducing behavioral cognitive changes.

## 2 Materials and methods

### 2.1 Bacterial cultures and preparation of OMVs

Pg strain ATCC 33277 was cultured on anaerobic agar plates supplemented with defibrillated sheep blood, brain-heart infusion (OXOID, Britain), hemin (0.5 mg/mL), and menadione (10 mg/mL) in an anaerobic system (Gene Science, America) with 10% CO_2_, 10% H_2_, and 80% N_2_. OMVs were isolated by an established protocol (Seyama et al., 2020). Briefly, when Pg cells were grown to the late exponential phase, the bacterial culture medium was collected and centrifuged at 2800 ×g for 15 minutes at 4°C to remove bacterial cells. The supernatant was filtered with a 0.22-μm syringe filter. The supernatant was concentrated to <1 mL by using an Ultra-15 Centrifugal Filter Device for nominal molecular weight limit (NMWL) 100,000 (Amicon, Merck, USA). The concentrate was mixed with Total Exosome Isolation Reagent (Invitrogen) and incubated overnight at 4°C. Afterward, the sample was centrifuged at 10,000 ×g for 60 minutes at 4°C. The OMV fractions were eluted with phosphate buffered saline (PBS) and quantified by protein concentrations using a BCA protein assay kit (Pierce, Rockford, IL, USA). Characterization of Pg OMVs utilized nanoparticle-tracking analysis (NTA) and transmission electron microscopy (TEM). To characterize the proteins of Pg OMVs, the same amount of Pg and OMVs (10 μg of total protein) were separated by sodium dodecyl sulfate (SDS)-poly-acrylamide gel electrophoresis (PAGE) and stained with Coomassie blue for one hour, washed in PBS overnight, and images were taken.

### 2.2 Animals

Fourteen-month-old male C57BL/6 mice were obtained from the Dashuo company. All animal studies were conducted following the guidelines approved by the Institutional Animal Care and Use Committee of the Stomatological Hospital of Chongqing Medical University. All mice were housed in groups of two to four mice per cage in biosafety barriers with a controlled light cycle and given sterile food and water ad libitum. The light–dark cycle was 1:1 with lights on at 7:00 a.m. Room temperature was 18°C  ±  2°C, and humidity was 55%  ±  10%. After one week of habituation, mice were treated with Pg OMVs or PBS by oral gavage using feeding needles (*n* = 12 in each group). The experimental group received Pg OMVs (4 mg/kg) diluted in PBS every other day for a consecutive eight weeks. The control group received an equivalent volume of PBS. The dosage of Pg OMVs were deduced from a previous study (Ho et al., 2016).

### 2.3 Mouse behavioral tests

A battery of behavioral tests comprising the Morris water maze (MWM), Y-maze, and open field tests were conducted to assess behavioral performance of mice.

#### 2.3.1 Morris water maze test

The MWM was conducted in a circular pool that was 120 cm in diameter, and it was filled with opaque water stained with milk and surrounded by a set of spatial cues. The tank was imaginarily divided into four quadrants. A platform that was 9 cm in diameter was submerged 1 cm under the water surface in a quadrant. The MWM test consisted of three platform trials per day for five consecutive days, followed by a probe trial. In the platform trial, the mouse navigated in the pool to locate the platform and was then able to escape. If the mouse failed to locate the platform within 60 seconds, it was directed to the platform. The mouse was allowed to remain on the platform for 60 seconds once it escaped onto the platform. The escape latency was measured to test spatial learning ability. In the probe trial, the platform was withdrawn. The mouse navigated freely in the pool for one minute. The time spent in each quadrant and the number of annulus crossings were recorded to assess memory consolidation.

#### 2.3.2 Y-maze test

A novel arm exploration test was performed in the Y-maze. One arm was blocked (defined as the novel arm), and the mice were allowed to explore the other two arms (home arm and familiar arm) for five minutes. After a one-hour interval, the mice were allowed to freely explore all three arms for five minutes. The number of novel arm entries and time spent in the novel arm were recorded.

#### 2.3.3 Open-field test

The mice were placed in the center of the open field apparatus for five minutes. The paths were tracked, and the distance travelled was recorded.

### 2.4 RNA isolation, reverse transcription, and quantitative real-time PCR

Total RNA was isolated by using the Trizol reagent (Takara, Japan), and it was subjected to reverse transcription by using the cDNA Reverse Transcription Kit (Takara, Japan). The quantitative real-time PCR analyses were carried out in the ABI Prism 7500 Real-Time PCR System (Applied Biosystems, Foster City, CA) with the SYBR Green PCR master mix reagent (Takara, Japan). PCR primer sequences are detailed in the supplementary data. The 2^-ΔΔCt^ value was used to calculate the relative gene expression normalized by the expression level of GAPDH.

### 2.5 Western blot assay

The hippocampus was homogenized and sonicated. The protein concentration was determined with a BCA protein assay reagent (Pierce, Rockford, IL, USA) according to the manufacturer’s instructions. The same amount of protein samples (30 μg) from each tube was boiled in 5 × loading buffer for 10 minutes at 95°C. The sample was then separated on 10% tris-glycine SDS-PAGE gels and transferred onto immobilon PTM polyvinylidene fluoride (PVDF) membranes. To block nonspecific background, the membranes were incubated in 5% nonfat milk in Tris-buffered saline containing 0.1% Tween-20 (TBST) at 37°C for one hour. The target proteins were immunoblotted with primary antibody overnight at 4°C (see the supplemental information for details) followed by incubation with a secondary antibody (1:1000; Cell signaling technology) at room temperature for one hour. The blots were imaged by the Bio-Rad Imager using ECL Western blotting substrate (Beyotime Technology). The relative level of target protein is expressed as the percentage between the intensity of the target protein and that of the marker protein, GAPDH. The band intensity of each protein was quantified by ImageJ software.

### 2.6 Brain sampling

The mice were killed by transcardial perfusion with saline after anesthetization. The left hemispheres were fixed in 4% paraformaldehyde (pH 7.4) for 24 hours, followed by incubation with 30% sucrose for 24 hours, and coronal sections were cut at 30 μm thickness for histological analysis. The right hemispheres were snap frozen in liquid nitrogen and stored at −80°C for Western blots and real-time PCR.

### 2.7 Immunofluorescence

Tissue sections were washed with PBS, penetrated with 0.5% Triton-100 for 20 minutes, blocked with normal serum, and incubated with primary antibodies (detailed in the supplemental information) at 4°C overnight. After PBS washing, anti-rabbit 488 (1:1000; Beyotime Technology) and anti-mouse 555(1:1000; Beyotime Technology) were added at 37°C for two hours. Then, the sections were incubated with DAPI (1:200; Beyotime Technology) and mounted in antifading medium (Beyotime technology), and images were acquired by confocal microscopy.

### 2.8 Tracking Pg OMVs in the brain

The OMVs (100 μg/mice) and the same volume of PBS were labeled with DiO (Beyotime Company, China) for 20 minutes at 37°C, fetal bovine serum was used to neutralize excessive dye, ultracentrifugation at 150,000 × g for one hour at 4°C was used to recollect labeled Pg OMVs, and then mice were treated with Pg OMVs or PBS by oral gavage. After three days, the mice were killed by transcardial perfusion with saline, and the brains were fixed in 4% paraformaldehyde for 24 hours, followed by incubation with 30% sucrose for 24 hours, and coronal sections were cut at 50-μm thickness, stained with DAPI, and viewed by confocal microscopy.

### 2.9 Cellular experiment

BV2 microglia cells and mouse neuroblastoma cell line N2a (kindly gifted by Dr. Zhifang Dong from Children’s Hospital of Chongqing Medical University) were maintained in high glucose Dulbecco’s modified Eagle’s medium (DMEM) supplemented with 10% fetal bovine serum, 100 units/ml penicillin, and 100 mg/ml streptomycin. For the vesicle uptake experiment, BV2 cells (1.5*10^4/^well) were seeded on glass cover slides and cultured in 12-well plates overnight. Pg OMVs (10 μg/ml) and saline were stained with PKH26 (Merck, USA) and then were added to the cultures and incubated for 30 minutes. After fixing with 4% paraformaldehyde at room temperature for 10 minutes and penetration with 0.1% Triton-100 for 10 minutes, the nuclear was stained with DAPI, and images were acquired by confocal microscopy. For BV2 and N2a stimulation experiments, the BV2 were stimulated with different concentrations of *Pg* OMVs, and 5 μg/ml was chosen for the rest of the *in vitro* experiments. BV2 were stimulated with Pg OMVs or pretreated with MCC950(NLRP3 inhibitor; 20 μM; AbMole) for one hour. After six hours of Pg OMV stimulation, the medium was changed to fresh medium, and the BV2 conditioned medium was harvested 24 hours later. The N2a was treated with the BV2 conditioned medium and harvested 24 hours later.

### 2.10 Statistical analyses

Two-way repeated measures ANOVA was used for escape latency in MWM measurements. The statistical analyses were performed by Student’s *t*-test and one-way ANOVA using the GraphPad Prism software package (GraphPad Software, California, USA). Data were expressed as means ± SEM, and a value of *p* <.05 was considered to indicate statistical significance.

## 3 Results

### 3.1 Characterization of Pg OMVs

**Figure 1:**
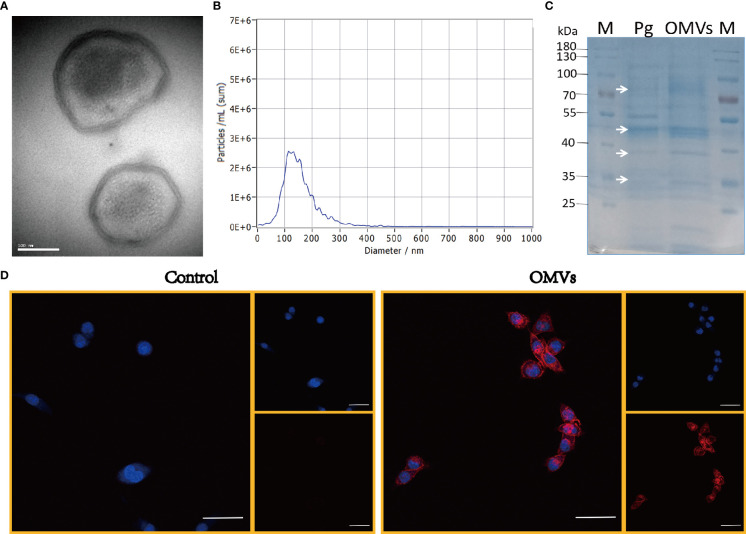
Characterization of Pg OMVs. **(A)** Pg OMVs observed by TEM. Scale bar = 100 nm. **(B)** NTA measured the diameter of Pg OMVs. **(C)** SDS-PAGE gel showed cargo proteins of Pg OMVs. White arrows indicate the bands in Pg OMVs samples (Pg OMVs) that correspond to Pg bacterial cell proteins (Pg). **(D)** Confocal images of Pg OMVs uptake by BV2 cells within 30 minutes, Nuclei (blue) were labeled with DAPI, Pg OMVs or PBS were labeled with PKH26 (red). Scale bar = 50 μm. TEM, transmission electron microscopy; NTA, nanoparticle-tracking analysis.

Pg OMVs were characterized by TEM, which revealed that Pg OMVs are spherical bilayered cell portions (**Figure 1A**), and NTA showed the average diameter of Pg OMVs was 115.7 ± 4.1 nm (**Figure 1B**), which is the typical OMV diameter of Gram-negative bacteria. To analyze the protein contents of Pg OMVs, Pg OMVs and Pg fractions were separated by SDS-PAGE and visualized by Coomassie blue. We found that some Pg OMV protein bands corresponded to those of the Pg bacterial cell, suggesting that Pg proteins were present in the OMVs (**Figure 1C**). The Pg OMVs could be internalized by BV2 microglia cells within 30 minutes (**Figure 1D**).

### 3.2 Effect of Pg OMVs on body weight and survival

**Figure 2:**
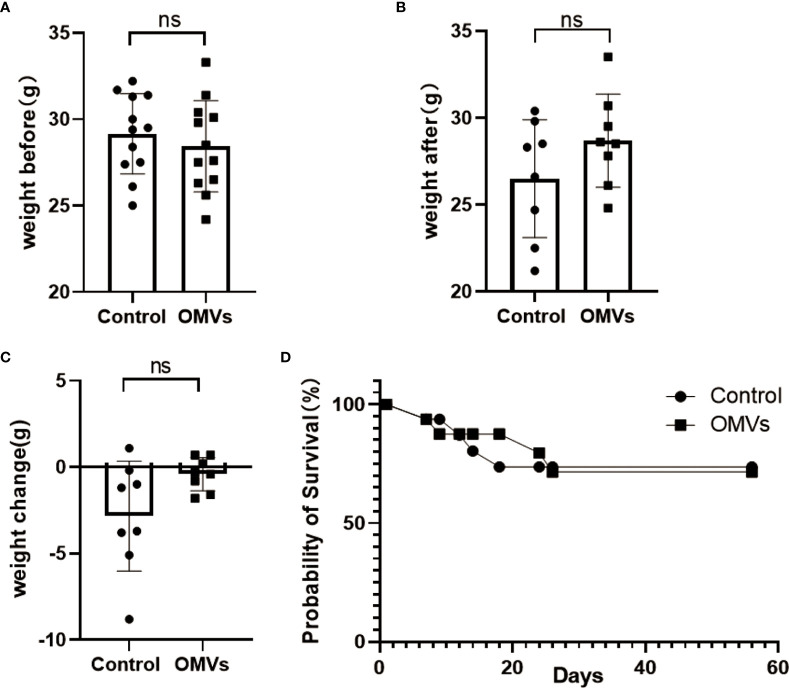
Pg OMVs did not affect body weight or survival rate. The mice were weighed before **(A)** and after **(B)** treatment, and the weight change **(C)** is shown. **(D)** Kaplan–Meier survival plots of mice. Data are presented as mean ± SEM. (ns: *P* >.05).

Twenty-four normal C57BL/6 mice, 14 months of age, were randomly divided into two groups: Pg OMVs and control. The mice were weighed before and after the eight‐week treatment, and the results were analyzed. There was no significant difference in the weight change (**Figures 2A–C**) or survival rates (**Figure 2D**) among the two groups before and after treatment.

### 3.3 Pg OMVs cross blood–brain barrier of mice and decrease tight junction–related gene and protein expression in the hippocampus of mice

**Figure 3:**
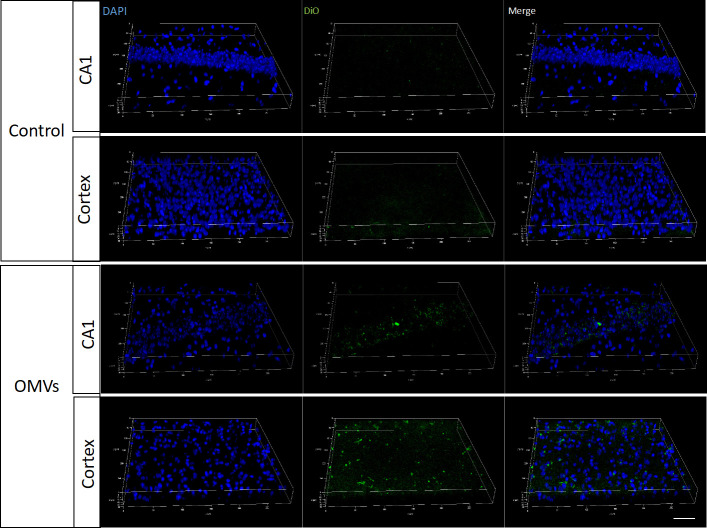
Pg OMVs enter the brain. Pg OMVs labeled with DiO (green) were applied by oral gavage for three days and were detected by confocal microscopy in the hippocampus and cortex of mice. Scale bar = 50 μm.

**Figure 4:**
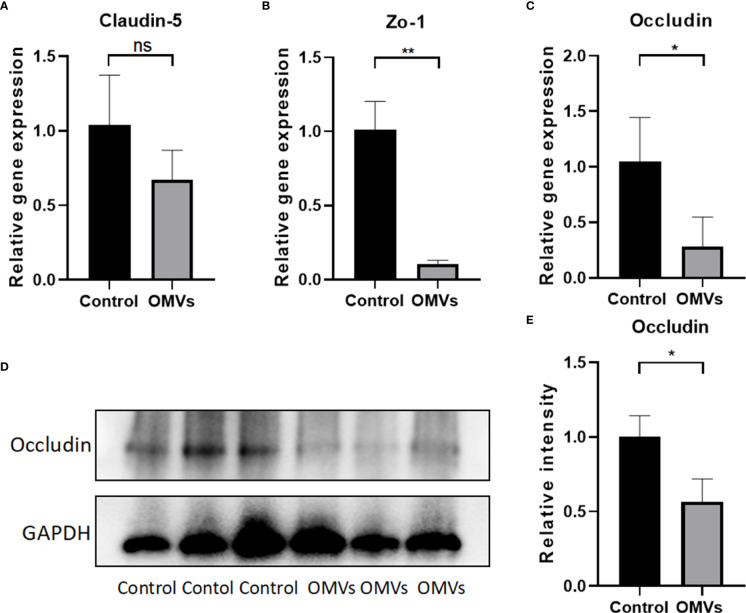
Oral gavage of Pg OMVs decreases tight junction–related gene and protein expression in the brain. **(A–C)** Gene expression of claudin-5, occluding, and zo-1 in the brain. **(D)** Occludin protein expression was measured by Western blot analysis. **(E)** The quantitative analysis of occludin immunoblots in **(D)**. Values are expressed as the mean ± SEM. OMVs, outer membrane vesicle; SEM, standard error of the mean. (*n* = 3, ns: *P* >.05, *p <.05, **p <.01).

To determine whether Pg OMVs could cross the blood–brain barrier, Pg OMVs labeled with DiO were applied to mice by oral gavage, and labeled Pg OMVs were clearly detected in the hippocampus and cortex after three days (**Figure 3**). To test whether Pg OMVs changed BBB permeability, we performed Western blot analysis and RT-qPCR to detect tight junction–related protein and gene expression after eight weeks oral gavage. Claudin-5, ZO-1, and occludin are important proteins for the tight junctions between capillary endothelial cells. RT-qPCR showed that claudin-5, ZO-1, and occludin gene expression were decreased in the hippocampus of Pg OMV–treated mice (**Figures 4A-C**). The Western blot indicated that occludin was significantly decreased in the hippocampus of Pg OMV–treated mice (**Figures 4D, E**).

### 3.4 Oral gavage of Pg OMVs impairs learning and memory ability in mice

**Figure 5:**
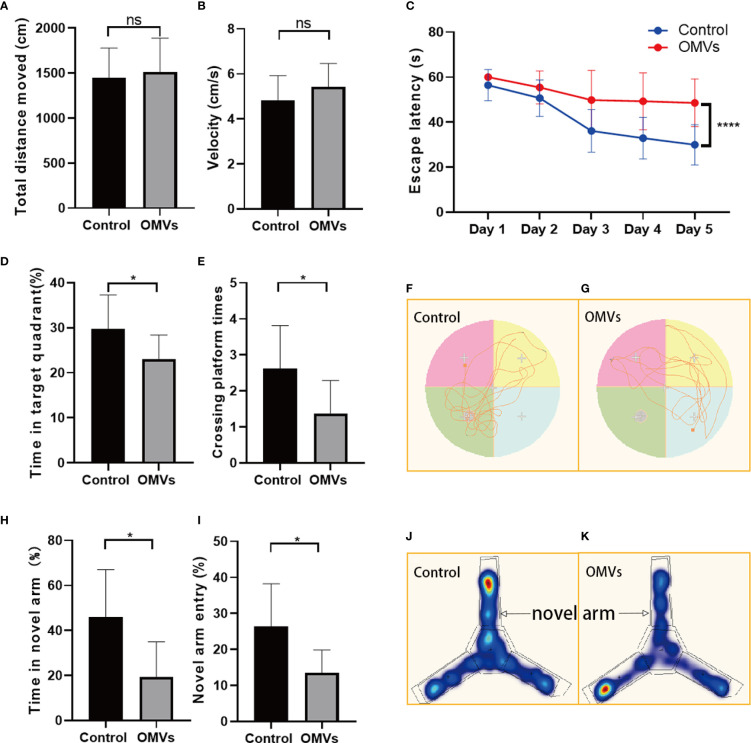
Oral gavage of Pg OMVs impairs memory and spatial learning of mice. **(A, B)** Distance and velocity traveled in the open-field test. **(C)** Latency to find the platform during the acquisition phase of the MWM test. **(D, E)** Crossing platform times and time in the target quadrant on the sixth day when the platform was removed. **(F, G)** Representative tracing graphs of Morris water maze on the sixth day. **(H, I)** Novel arm entry and time spent in the novel arm in the Y-maze test. **(J, K)** Representative hot spot graphs. (*n* = 8, ns: *P* >.05, *p <.05, ****p <.0001).

Sickness may reduce local motor activity, which may affect mouse behavior in the MWM and Y-maze tests. To exclude the effects of sickness symptoms on mouse behavior, the open field test was performed. In open field test, there was no significant difference in traveling distance or velocity between the two groups (**Figures 5A, B**), suggesting that Pg OMVs did not impair the locomotor activity of mice. To test whether Pg OMVs had an effect on memory and learning ability, we performed the MWM and Y-maze tests. In the MWM test, Pg OMVs impaired learning and spatial reference memory in mice as reflected by an increase in the escape latency in the five-day platform trial (**Figure 5C**). In the probe trial on the sixth day, the Pg OMV–treated mice showed worse memory consolidation as reflected by a lower number of platform area crossings and less time spent in the target quadrant compared with controls (**Figures 5D, E**) representative tracing graphs of Morris water maze on the sixth day were shown in **Figures 5F, G**. In the Y-maze test, the Pg OMV–treated mice showed a significantly decreased number of entries and time spent in the novel arm (**Figures 5H–K**).

### 3.5 Oral gavage of Pg OMVs increases tau phosphorylation in the brain of mice

**Figure 6:**
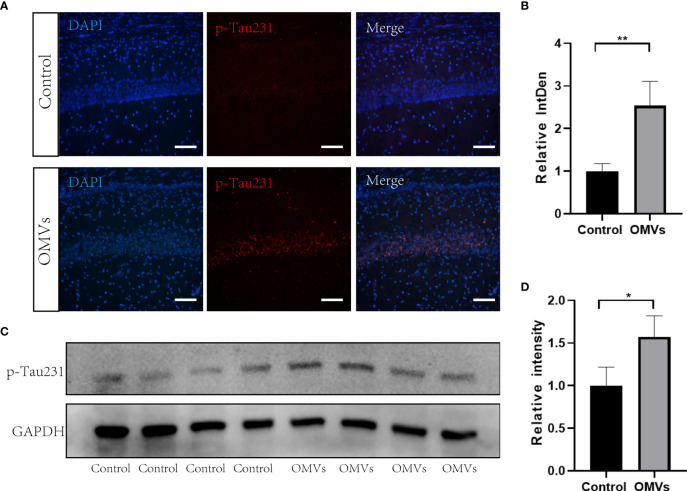
Oral gavage of Pg OMVs increases tau phosphorylation in the hippocampus of mice. **(A)** Immunofluorescent images of p-Tau231 (red) in the hippocampus of mice. Scale bar = 50 μm. **(B)** Mean relative fluorescence intensity (relative integrated density) of p-Tau231 in image **(A)** (*n* = 3, **p <.01, Student’s *t*-test). **(C)** The Western blots showing p-Tau231 in the hippocampus of mice. **(D)** The quantitative analyses of the ratio of p-Tau231 immunoblots in the image **(C)** (*n* = 4, *p <.05).

Tau phosphorylation is one of the hallmark pathologies in AD and is closely related to memory consolidation. Immunofluorescent images showed that the Thr231-site phosphorylation in the hippocampus was significantly increased in the experimental group (**Figures 6A, B**), and the mean degree of tau phosphorylation on the Thr231 site was increased significantly in the hippocampus of mice in the experimental group (**Figures 6C, D**).

### 3.6 Oral gavage of Pg OMVs induces neuroinflammation

**Figure 7:**
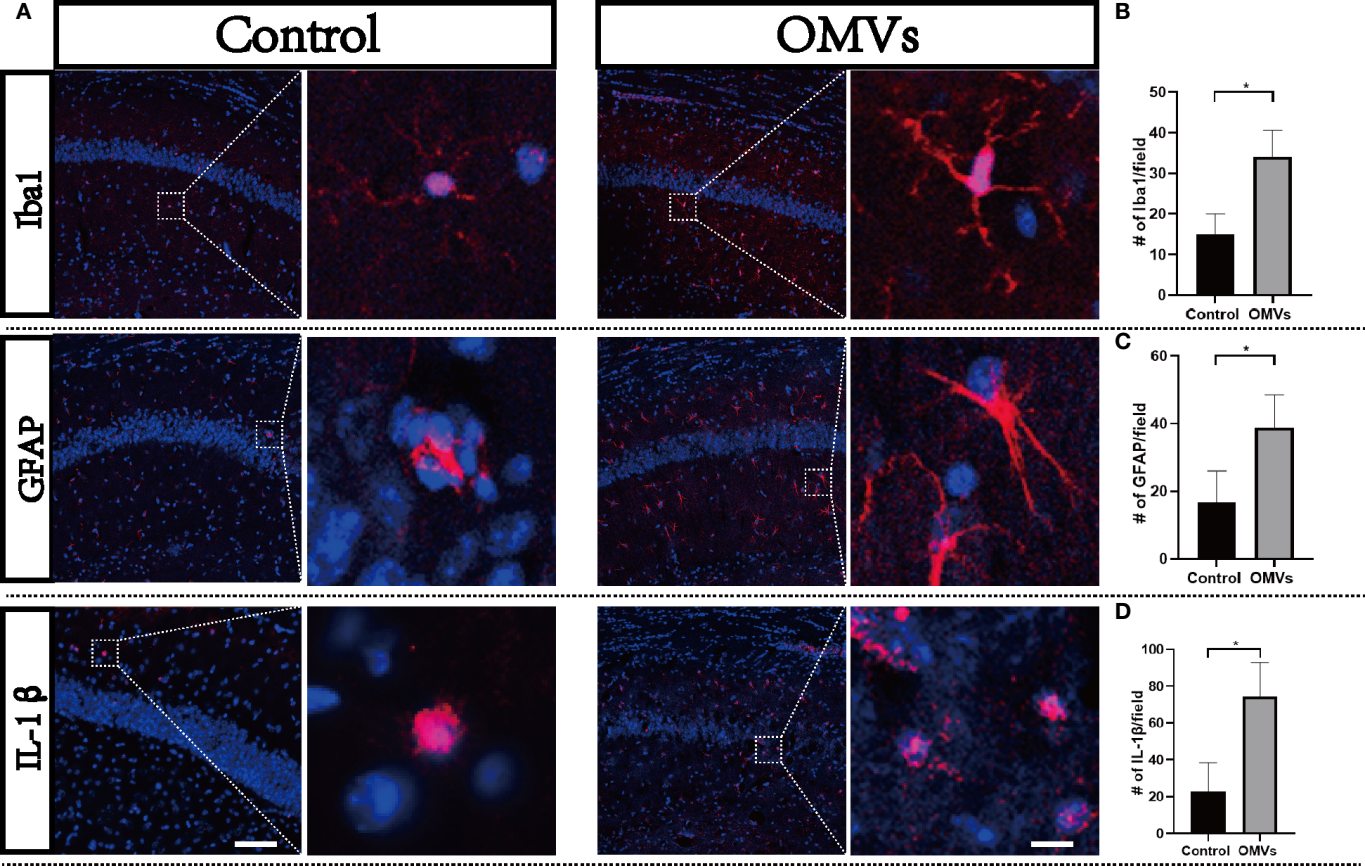
Oral gavage of Pg OMVs induces neuroinflammation. **(A)** Activation of microglia (Iba1 marker, red) and astrocytes (GFAP marker, red) and expression of IL-1β (red) in the hippocampus of mice. Scale bar left = 50 μm, scale bar right = 5 μm. **(B)** Number of microglia (Iba1 positive) counted in the hippocampus from five slides for each mouse and are means ± SEM; *n* = 3 mice/group. **(C)** Number of astrocytes (GFAP positive) in the hippocampus counted from five slides for each mouse and are means ± SEM; *n* = 3 mice/group. **(D)** Number of IL-1βpositive cells in the hippocampus counted from five slides for each mouse and are means ± SEM; *n* = 3 mice/group. *p <.05.

To clarify whether Pg OMVs could induce neuroinflammation, we detected microglia, astrocytes, and IL-1β by immunofluorescence. Microglia are resident macrophage-like cells in the brain, which would be overactivated and release proinflammatory cytokines in response to microbial infection. The immunofluorescent images showed that a number of Iba1-positive microglia were significantly increased in the experimental group (**Figures 7A, B**). Astrocytes are known to function in biochemical support of endothelial cells that form the BBB. An increased number of astrocytes can be induced by infection. We then examined whether astrocytes in the hippocampus were activated by using a GFAP antibody. There was a significantly higher number of astrocytes in the experimental group compared with controls (**Figures 7A, C**). IL-1β played a central role in neuroinflammation, and the number of IL-1β positive cells were significantly increased in the experimental group (**Figures 7A, D**).

### 3.7 Oral gavage of Pg OMVs activates the NLRP3 inflammasome in the hippocampus of mice

**Figure 8:**
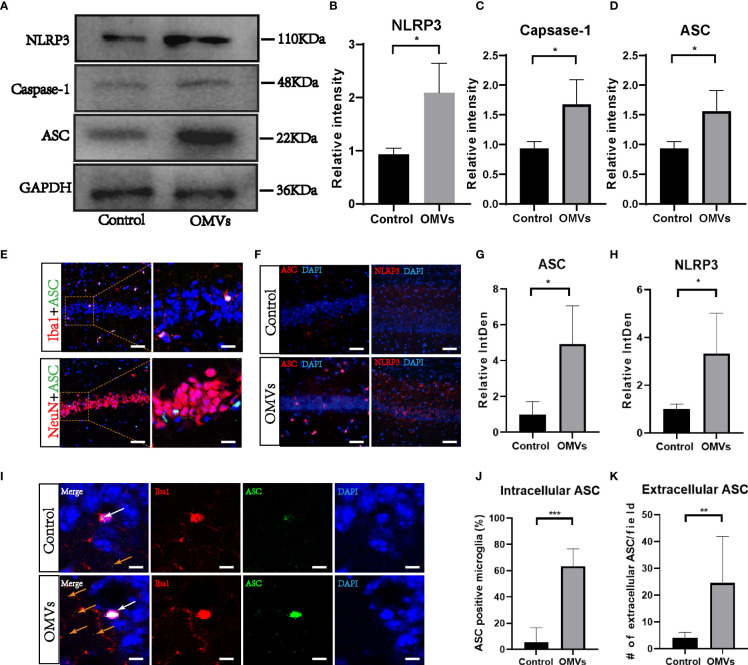
Oral gavage of Pg OMVs triggers NLRP3 inflammasome in the hippocampus of mice. **(A)** The Western blots showed NLRP3, caspase-1, and ASC in the hippocampus of mice. **(B–D)** The quantitative analyses of immunoblots in image **(A)** (*n* = 3, *p <.05). **(E)** Co-localization of ASC with neuronal cell marker (NeuN) and microglia marker (Iba1) revealed expression of ASC in microglia but not in neurons, scale bar left = 50 μm, scale bar right = 20 μm. **(F)** Immunofluorescent images showed ASC (red) and NLRP3 (red) expression in hippocampus of mice, scale bar = 50 μm. **(G, H)** The quantitative analyses of immunoblots in image **(F)** (*n* = 3, *p <.05) **(I)** Immunofluorescent images showed intracellular ASC (white arrow) and extracellular ASC (yellow arrows) in hippocampus of mice. Scale bar = 5 μm. **(J)** The percentage of intracellular ASC positive microglia in hippocampus of mice, counted from five slides per mouse and expressed as means ± SEM; *n* = 4 mice/group. (*n* = 4, ***p <.001) **(K)** Number of extracellular ASC per field in hippocampus of mice. (*n* = 4, **p <.01).

Since the NLRP3 inflammasome is reported to drive microglia activation and tau pathology (Ising et al., 2019; Lempriere, 2020), we next explored whether the NLRP3 inflammasome was activated. Proteins of NLRP3, ASC, and caspase-1 were elevated significantly in the hippocampus of the experimental group (**Figures 8A–D**). Immunofluorescence double staining showed that ASC co-localized mainly with microglia (**Figure 8E**; **Figures S1, 2**). Immunofluorescent images showed that the fluorescent intensity of ASC and NLRP3 were significantly increased in the experimental group in the hippocampus (**Figures 8F–H**), and intracellular and extracellular ASC specks were significantly increased in the experimental group (**Figures 8I–K**).

### 3.8 Pg OMVs activate the NLRP3 inflammasome in cultured microglia and contributes to tau hyperphosphorylation in cultured neurons

**Figure 9:**
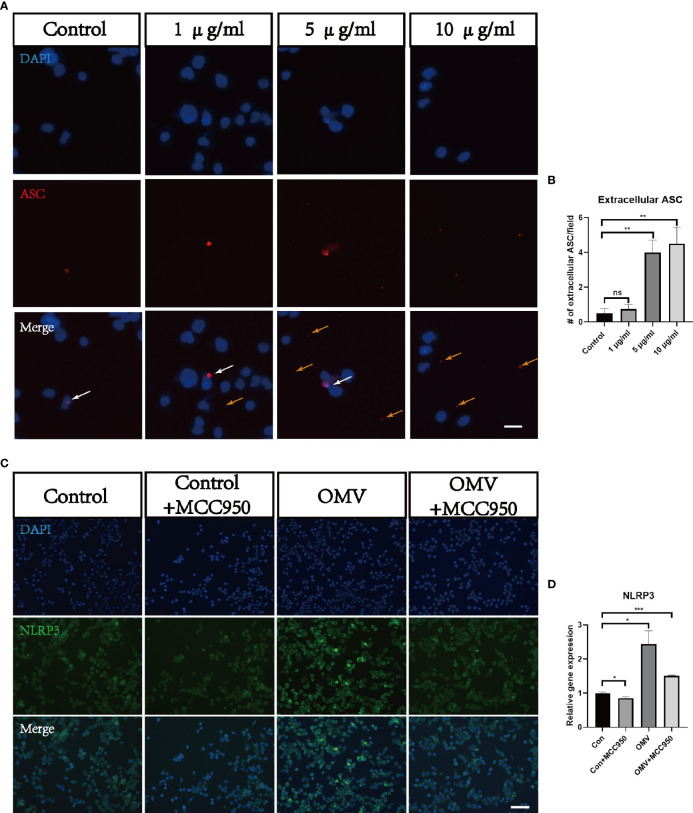
Pg OMVs triggers NLRP3 inflammasome in microglia *in vitro*. **(A)** Immunofluorescent images showed intracellular ASC (white arrow) and extracellular ASC (yellow arrows) of BV2 cells stimulated with different concentrations of Pg OMVs. Scale bar = 10 μm. **(B)** Number of extracellular ASC per field. (*n* =4, ns, not significant, **p <.01). **(C)** Immunofluorescent images showed NLRP3 staining of BV2 cells stimulated by Pg OMVs. MCC950 (20 μM) were used for pretreatment for one hour. **(D)** The mRNA expression of NLRP3 in BV2 cells stimulated by Pg OMVs. MCC950 (20 μM) were used for pretreatment for one hour. (n=3, *p <0.05).

**Figure 10:**
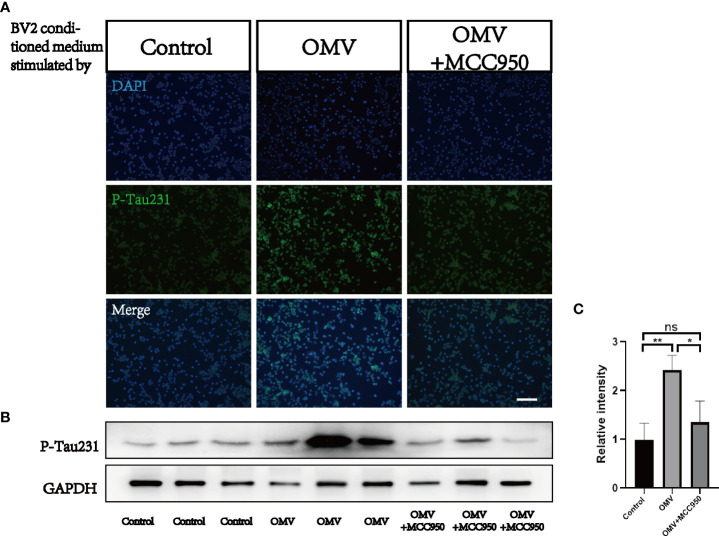
Microglia-conditioned medium (MCM) stimulated by Pg OMVs contributes to tau hyperphosphorylation in cultured neurons. **(A)** Immunofluorescent images of P-Tau231 in N2a cells treated with MCM from control, Pg OMV–primed MCM, or Pg OMV–primed MCM pretreated with NLRP3 inhibitor for 24 hours. **(B)** The Western blot images of P-Tau231 in N2a cells treated with MCM from control, Pg OMV–primed MCM, or Pg OMV–primed MCM pretreated with NLRP3 inhibitor for 24 hours, samples from three independent experiments are shown. **(C)** The quantitative analyses of immunoblots in image **(B)** (*n* = 3, *p <.05, **p <.01). (ns: p >.05).

To test whether Pg OMVs activate the NLRP3 inflammasome and promote tau phosphorylation *in vitro*, we first measured extracellular ASC specks of BV2 cells with Pg OMV stimulation. The result showed that the extracellular ASC specks increased in a Pg OMV–dosage manner (**Figures 9A, B**), the 5 μg/ml dosage of Pg OMVs was chosen in the rest of the *in vitro* experiments. The expression of NLRP3 was increased with Pg OMV stimulation and decreased when pretreated with the NLRP3 inhibitor MCC950 (**Figures 9C, D**). To clarify whether the effects of Pg OMVs on tau phosphorylation in neurons are exerted indirectly *via* microglial activation, we treated N2a neurons with microglia-conditioned medium (MCM). Compared with MCM from control, a significantly increased mean degree of phosphorylated tau at Thr231 (**Figures 10A–C**) was observed in the N2a neurons following incubation with MCM stimulated by Pg OMVs. In contrast, the expression of phosphorylated tau at Thr231 was significantly decreased in the N2a neurons following incubation with NLRP3 inhibitor–pretreated and Pg OMV–treated MCM.

## 4 Discussion

The present study indicates that chronic infection by oral gavage of Pg OMVs induced AD-like phenotypes, including learning and memory deficiency, microglia-mediated neuroinflammation, and intracellular tau phosphorylation. NLRP3 inflammasome activation is involved in this process. To the best of our knowledge, this study, for the first time, demonstrates the relationship between Pg OMVs and AD-like phenotypes.

There are studies showing Pg LPS (Poole et al., 2013) and Pg DNA (Emery et al., 2017; Dominy et al., 2019) in autopsy specimens from brains of AD patients. Gingipains, the toxic protease of Pg, was also detected in the brain of AD patients, and levels correlated with tau pathology (Dominy et al., 2019). *In vivo* studies identified gingipains in the neuron, microglia, and astrocytes after oral application of Pg for 22 weeks in mice (Ilievski et al., 2018). However, none of these studies recovered Pg in the brains of AD patients or in the brains of mice, leaving the question of whether live Pg or just its virulence factors entered the brain. We focused on Pg OMVs because Pg OMVs contain LPS, DNA, and gingipains that were detected in the brain of AD patients. Compared with Pg, Pg OMVs have advantages to enter the brain. First, they are greater in quantity. The number of Pg OMVs is about 2000 times of the number of Pg (Fleetwood et al., 2017). Second, they are smaller in size; the diameter of Pg OMVs is about 80 nm on average, whereas the diameter of Pg is about 600 nm (Gui et al., 2016), which is seven times larger than that of Pg OMVs. Third, Pg OMVs contain gingipains and LPS, which are reported to cause pathological changes related to AD. Compared with the same protein amount of Pg, Pg OMVs are reported to contain three to five times the gingipains compared with that of Pg (Mantri et al., 2015). Last but not least, Pg OMVs invade epithelial cells more efficiently than Pg (Ho et al., 2015). Thus, we speculate that Pg OMVs are important virulence factors of Pg in the relationship between Pg and AD.

The first question is whether Pg OMVs can cross the BBB and enter the brain. We detected Pg OMVs in the cortex and hippocampus of mice three days after oral gavage. This is in agreement with Lee et al. In their study, five days after oral gavage of extracellular vesicles of Paenalcaligenes hominis, a member of the *Proteobacteria* in the gut, the extracellular vesicles could be detected in the hippocampus of mice, and vagotomy inhibited the accumulation of Paenalcaligenes hominis extracellular vesicles in the hippocampus (Lee et al., 2020). OMVs of *Aggregatibacter actinomycetemcomitans*, a well-known periodontal pathogen, can cross BBB and be detected in the brain 24 hours after cardiac injection (Han et al., 2019) or intravenous injection (Ha et al., 2020). OMVs of gut microbiota could be detected in the hippocampus 12 hours after intravenous injection (Wei et al., 2020). Bittel et al. found that OMVs of *Escherichia coli* could be detected in the brain after oral gavage of *Escherichia coli*, but *Escherichia coli* could not be detected in the brain. This is the first proof-of-principle study visualizing that OMVs, but not the parental bacteria could transport to the brain from the gut (Bittel et al., 2021).

The BBB forms an effective barrier between the central nervous system and the blood to maintain homeostasis of the brain microenvironment (Keaney and Campbell, 2015). A correlation between BBB dysfunction and tau pathology is reported (Cai et al., 2018). Tight junctions, consisting of cytoplasmic proteins (ZOs), transmembrane proteins (occludin and claudins), and cytoskeleton proteins, play a critical role in maintaining the integrity and permeability of the BBB (Zihni et al., 2016). We found that Pg OMVs decreased ZO-1 and occludin gene expression and also decreased occludin protein expression, suggesting that Pg OMVs caused disruption of the BBB, which would facilitate Pg OMVs and other harmful compounds in the blood to enter the brain. OMVs of gut microbiota were also reported to decrease tight junctions of the BBB (Wei et al., 2020). *In vitro*, Pg OMVs could induce degradation of ZO-1 and occludin in human cerebral microvascular endothelial cell lines, and gingipains in Pg OMVs played a critical role in tight junction degradation (Nonaka et al., 2022).

In our study, chronic oral gavage of Pg OMVs did not influence body weight or locomotion, suggesting that Pg OMV–induced learning and memory impairment was not associated with sickness behaviors. Tau pathology is closely linked to learning and memory functions in AD pathology (van der Kant et al., 2020). Tau phosphorylation on the Thr231 site is shown to be significantly increased in the early stage of AD (Neddens et al., 2018). We show that Pg OMVs could increase tau phosphorylation on the Thr231 site in the hippocampus of mice, which may be caused by gingipains of Pg OMVs. The abundance of gingipains correlates with the tau protein load in AD brain autopsies (Dominy et al., 2019), and gingipains can cleave tau *in vitro* (Dominy et al., 2019), an activity that might contribute to aberrant tau phosphorylation and accumulation of insoluble tau forms in AD (Kovacech and Novak, 2010). Other studies also demonstrate that Pg or Pg LPS can elevate tau phosphorylation. Tang et al. intravenously injected Pg for 12 weeks and found that tau phosphorylation on the Thr231 and Thr181 sites were increased in the hippocampus of rats (Tang et al., 2021), and Jiang et al. peritoneally injected Pg LPS for three weeks in APP^NL-F/NL-F^ mice and found that tau phosphorylation on the Ser202, Thr231, and Ser396 sites were elevated in the cortex of mice (Jiang et al., 2021). Ilievski et al. orally applied Pg for 22 weeks in mice and detected that tau phosphorylation on the Ser396 site was increased in the hippocampus of mice (Ilievski et al., 2018).

Microglia-mediated neuroinflammation is a key factor involved in regulating AD pathogenesis, and IL-1β is associated with the progression and onset of AD (Alvarez et al., 1996; Oprica et al., 2007; Deniz-Naranjo et al., 2008). Since Pg OMVs could activate the NLRP3 inflammasome and induce IL-1β in macrophages (Cecil et al., 2017), we speculate that NLRP3 inflammasome activation is involved in the process. We found that NLRP3 and ASC were significantly elevated in the hippocampus of mice. ASC mainly co-localized with microglia, and few co-localized with neurons (**Figure S1**), which is in accordance with other studies (Lei et al., 2017; Reimers et al., 2021). ASC specks were reported to be principally in the nuclei of microglia with a small amount in microglia processes (Reimers et al., 2021). Since inflammasome activation results in the activation of highly pro-inflammatory cytokines and the death of the activated cell, ASCs specks could be found in the extracellular space after cell death. In fact, extracellular ASC specks were reported to have prionoid activities and further promote IL-1β maturation (Franklin et al., 2014). We found that both intracellular and extracellular ASC specks were increased in the experimental group, which suggested an inflammatory state. Overexpression of IL-1β exacerbates tau phosphorylation and tangle formation (Sheng et al., 2000; Kitazawa et al., 2011), which affects synaptic plasticity, inhibiting long-term potentiation and, subsequently, learning and memory (Pickering and O'Connor, 2007).

Oral gavage of Pg OMVs was applied in this study. Ding et al. applied Pg by oral gavage to middle-aged mice and induced AD-like pathologies (Ding et al., 2018). We speculate that oral gavage of Pg OMVs would change gastrointestinal microbiota and, thus, indirectly influence AD-like pathologies since gastrointestinal microbiota were reported to be related to AD (Pistollato et al., 2016, Doulberis et al., 2019, Papaefthymiou et al., 2019, Giovannini et al., 2021). In fact, according to a quantitative analysis, it was estimated that patients with severe periodontitis swallowed approximately 10^12^~10^13^ Pg per day (Boutaga et al., 2007; Saygun et al., 2011; He et al., 2012), oral gavage of Pg once or for five weeks in mice have been reported to change gut microbiota with Bacteroidetes increased and Firmicutes decreased, indicating an inflammatory state (Nakajima et al., 2015; Kato et al., 2018). Ligature-induced periodontitis is reported to change gut microbiota and associated with memory decline in mice, and tight junctions of the gut and brain were decreased (Xue et al., 2020).

The current study is beset with certain limitations. First, the dosage and duration of Pg OMVs treatment should be investigated in further study. Second, oral gavage of Pg OMVs may not fully simulate the pathology of periodontitis. Last, the effect of Pg OMVs compared with Pg in inducing AD-like pathology is not explored in this study. Our group is conducting relevant studies now to further deduce the effect of Pg OMVs in Pg related to AD. However, this study is a novel attempt, and we believe that OMVs are important weapon of bacteria and expect the current study to draw readers’ attention to OMVs.

**Figure 11:**
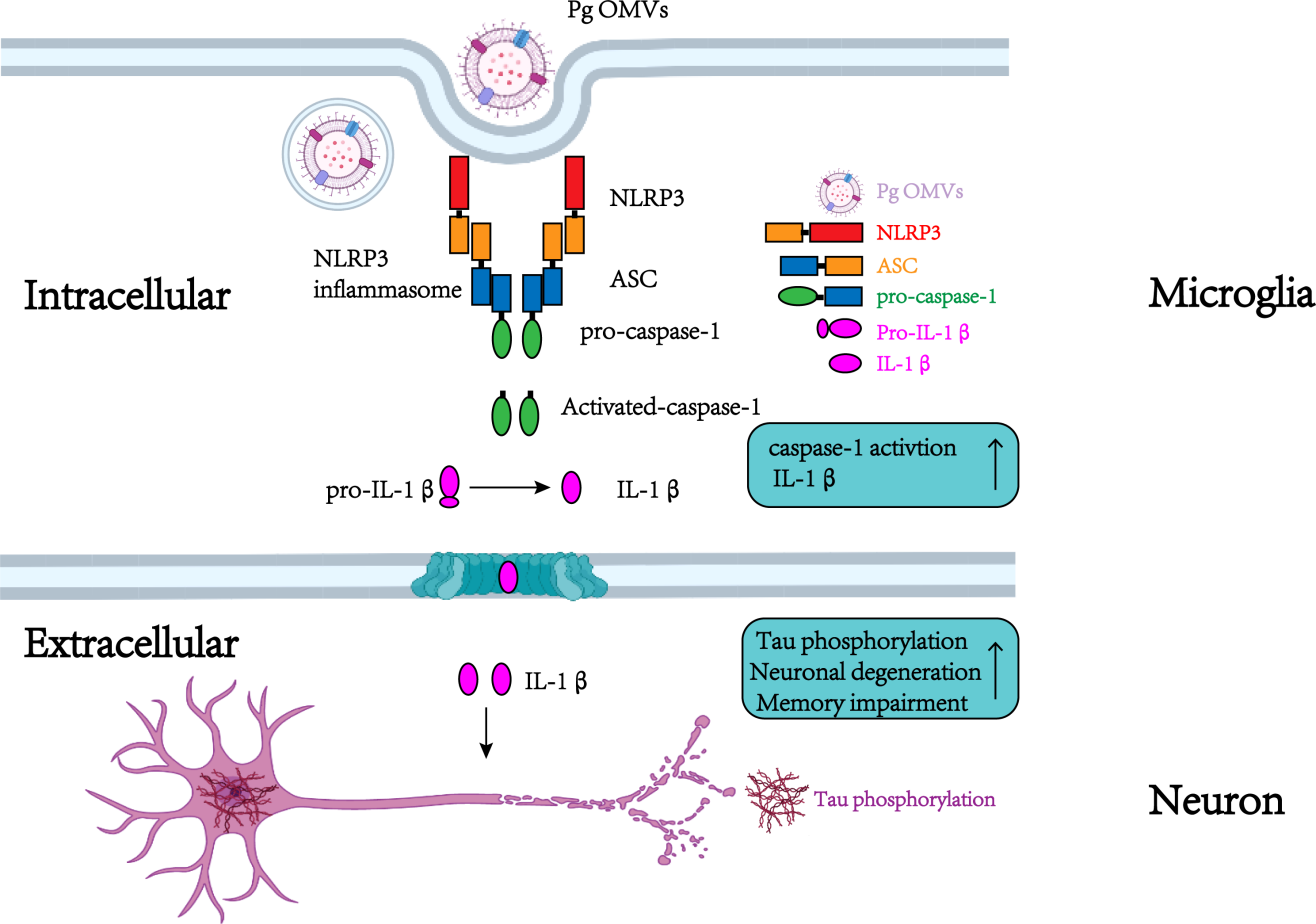
Schematic to show the roles of Pg OMVs on microglia and neurons. In microglia, Pg OMV induce IL-1β production though the activation of the NLRP3 inflammasome. In neurons, the microglia-released IL-1β is involved in tau phosphorylation, neuronal degeneration, and memory impairment. Created with BioRender.com.

In conclusion, we reveal for the first time that Pg OMVs promote the activation of astrocytes and microglia, activate NLRP3 inflammasome, elevate IL-1β and tau phosphorylation, which further impairs the cognitive function (**Figure 11**).

## Data availability statement

The original contributions presented in the study are included in the article/**Supplementary Files**, further inquiries can be directed to the corresponding author/s.

## Ethics statement

The animal study was reviewed and approved by the Institutional Animal Care and Use Committee of the Stomatological Hospital of Chongqing Medical University.

## Author contributions

TG wrote the main manuscript text. DY critically revised Manuscript. TG, QC, HM, YZ, HR, MX and HC performed the research. TG, QC, HM, YZ, HR, MX, HC and DY analyzed the data. TG and DY designed the main research study and provided the necessary guidance on the performance of all the experiment. TG, HC and DY contributed the essential reagents or tools. All authors read and approved the final manuscript.

## Funding

This work was sponsored by Program for Youth Innovation in Future Medicine, Chongqing Medical University (No.W0060 to DY), Program for Top talent Distinguished Professor from Chongqing Medical university (No.[2021]215 to DY), Natural Science Foundation of Chongqing, China (No.cstc2021jcyj-bshX0176 to TG), Chongqing Special Postdoctoral Science Foundation (No. 2010010005994583 to HC).

## Conflict of interest

The authors declare that the research was conducted in the absence of any commercial or financial relationships that could be construed as a potential conflict of interest.

## Publisher’s note

All claims expressed in this article are solely those of the authors and do not necessarily represent those of their affiliated organizations, or those of the publisher, the editors and the reviewers. Any product that may be evaluated in this article, or claim that may be made by its manufacturer, is not guaranteed or endorsed by the publisher.
