# Supplementary material for: Outer membrane vesicles of Porphyromonas gingivalis trigger NLRP3 inflammasome and induce neuroinflammation, tau phosphorylation, and memory dysfunction in mice
Source: Front Cell Infect Microbiol. 2022 Aug 9;12:925435. doi: 10.3389/fcimb.2022.925435 (PMC9397999; doi:10.3389/fcimb.2022.925435)

Table1.Primer sequences used for quantitative polymerase chain reaction evaluation of gene expression.

| Name | Forward | Reverse |
| --- | --- | --- |
| ZO-1 | 5’-AGCGAAAACCCGAAACTGATG-3’ | 5’-TGATACTGAGTTGCCTTCACCCT-3’ |
| Claudin-5 | 5’-TCGGGTGAGCATTCAGTCTTTAG-3’ | 5’-ACGACATCCACAGCCCCTTC-3’ |
| Occludin | 5’-ATAATGGGAGTGAACCCGACG-3’ | 5’-CCACGATAATCATGAACCCCA-3’ |
| NLRP3 | 5’-TCCACAATTCTGACCCACAA-3’ | 5’-ACCTCACAGAGGGTCACCAC-3’ |

Table 2. Primary antibodies used in this study.

| Antibody | Company | Application | Host | Dilution |
| --- | --- | --- | --- | --- |
| Occludin | Santa cruz, USA, SC-133256 | WB | Mouse | 1:1000 |
| GAPDH | Beyotime, China, AF0006 | WB | Mouse | 1:1000 |
| NLRP3 | Cell signaling, USA, 20836T | WB | Rabbit | 1:1000 |
| Caspase-1 | Cell signaling, USA, 20836T | WB | Rabbit | 1:1000 |
| Tau Thr231 | Cell signaling, USA, 71429 | WB/IF | Rabbit | 1:1000,1:100 |
| Iba1 | Cell signaling, USA, 17198T | IF | Rabbit | 1:200 |
| Iba1 | Proteintech, China, 66827-1-Ig | IF | Mouse | 1:100 |
| GFAP | Cell signaling, USA, 3670S | IF | Mouse | 1:300 |
| IL-1β | Cell signaling, USA, 20836T | IF | Rabbit | 1:100 |
| ASC | Cell signaling, USA, 20836T | IF | Rabbit | 1:100 |
| NeuN | Proteintech, China, 66836-1-Ig | IF | Mouse | 1:100 |
| NLRP3 | Proteintech, China, 19771-1-AP | IF | Rabbit | 1:100 |

IF, Immunofluorescence; WB, Western blot.

Fig. S1. Immunofluorescence double staining showed that ASC co-localized with microglia. a-d. Representative image of cortex and hippocampus. e. Image of D without DAPI. f. Image of D in yellow square. Scale bar= 50 μm.


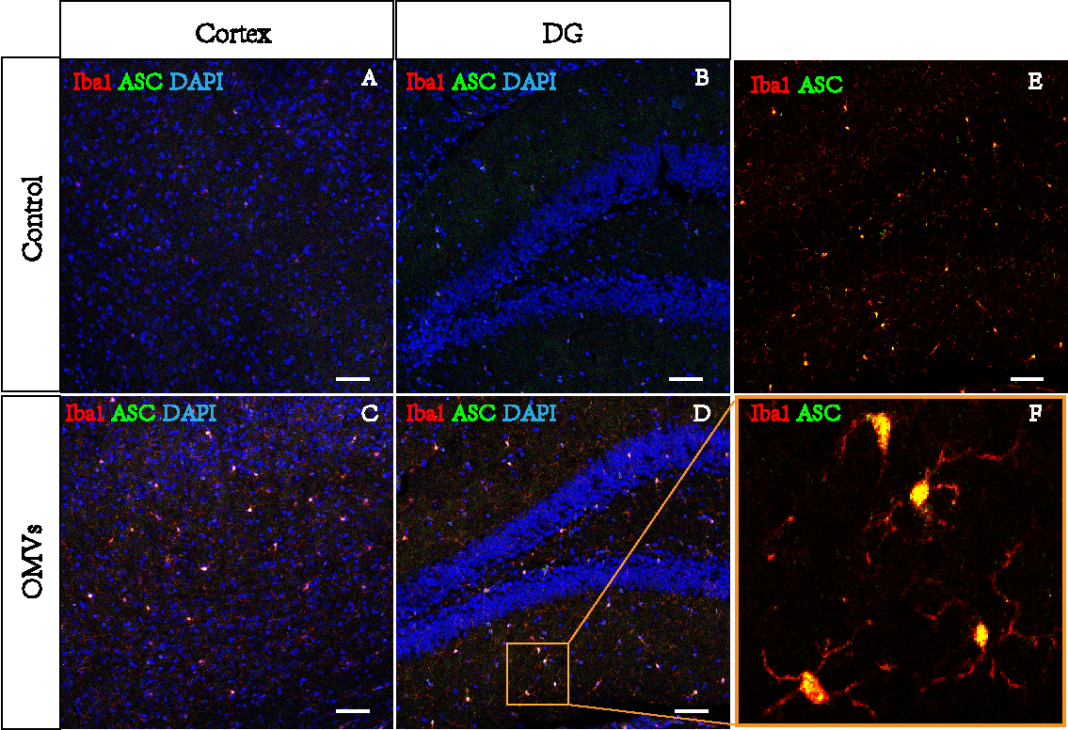


Fig. S2. Immunofluorescence double staining of ASC and NeuN. a-d. Representative image of cortex and hippocampus. e. Image of D without DAPI. f. Image of D in yellow square. Scale bar= 50 μm.


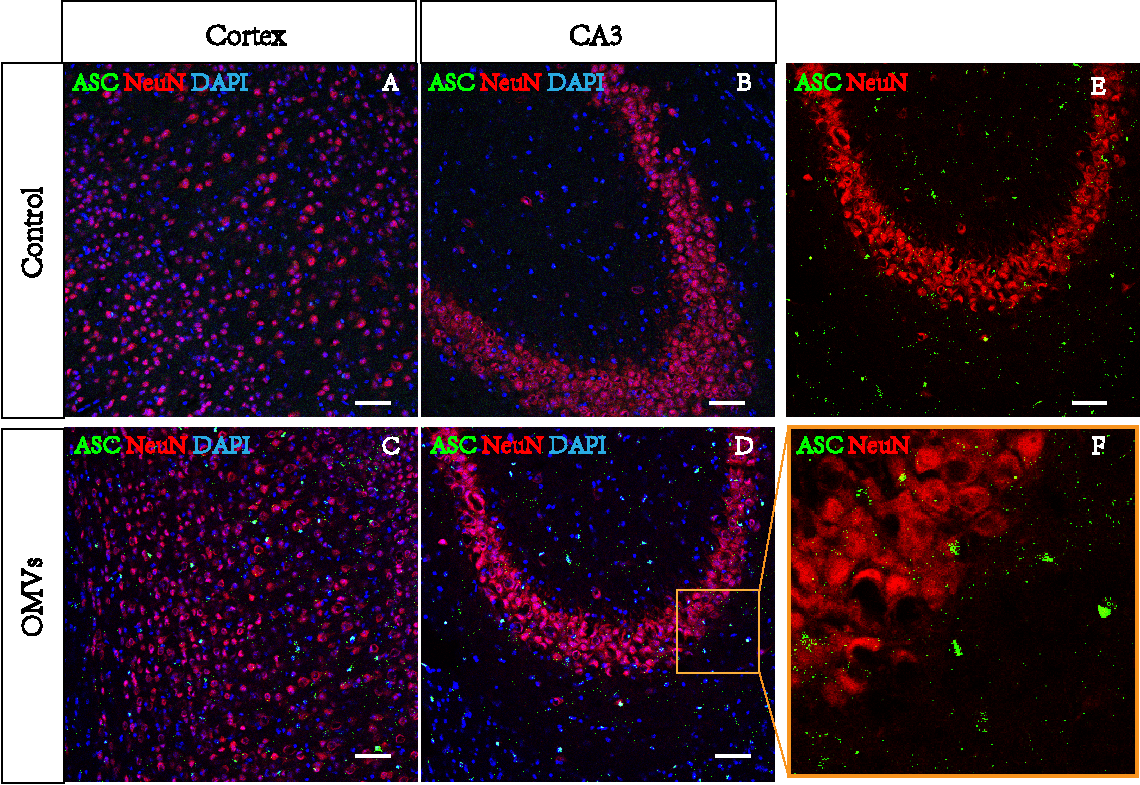

Supplement: Supplementary file 1 [file DataSheet_1.docx]
